# Near-miss organizational learning in nursing within a tertiary hospital: a mixed methods study

**DOI:** 10.1186/s12912-022-01071-1

**Published:** 2022-11-16

**Authors:** Tingting Feng, Xin Zhang, Lingling Tan, Yuanyuan Su, Huaping Liu

**Affiliations:** 1 School of Nursing, Chinese Academy of Medical Sciences & Peking Union Medical College, 33 Ba Da Chu Rd. Shijingshan District, 100144 Beijing, China; 2 Department of Nursing, The Second Affiliated Hospital of University of South China, Hengyang, Hunan Province China; 3 School of Nursing, The University of South China, Hengyang, Hunan Province China

**Keywords:** Near-miss, Organizational learning, Nursing organization, Mixed methods study

## Abstract

**Background:**

Near-miss organizational learning is important for perspective and proactive risk management. Although nursing organizations are the largest component of the healthcare system and act as the final safety barrier, there is little research about the current status of near-miss organizational learning. Thus, we conducted this study to explore near-miss organizational learning in a Chinese nursing organization and offer suggestions for future improvement.

**Methods:**

This was a mixed methods study with an explanatory sequence. It was conducted in a Chinese nursing organization of a tertiary hospital under the guidance of the 4I Framework of Organizational Learning. The quantitative study surveyed 600 nurses by simple random sampling. Then, we applied purposive sampling to recruit 16 nurses across managerial levels from low-, middle- and high-scored nursing units and conducted semi-structured interviews. Descriptive statistics, structured equation modelling and content analysis were applied in the data analysis. The Good Reporting of A Mixed Methods Study (GRAMMS) checklist was used to report this study.

**Results:**

Only 33% of participants correctly recognized near-misses, and 4% of participants always reported near-misses. The 4I Framework of Organizational Learning was verified in the surveyed nursing organization (χ^2^ = 0.775, *p* = 0.379, RMSEA < 0.01). The current organizational learning behaviour was not conducive to near-miss organizational learning due to poor group-level learning (*β*_GG_ = 0.284) and poor learning absorption (*β*_Misalignment_= -0.339). In addition, the researchers developed 13 codes, 9 categories and 5 themes to depict near-miss organizational learning, which were characterized by nurses’ unfamiliarity with near-misses, preferences and the dominance of first-order problem-solving behaviour, the suspension of near-miss learning at the group level and poor learning absorption.

**Conclusion:**

The performance of near-miss organizational learning is unsatisfactory across all levels in surveyed nursing organization, especially with regard to group-level learning and poor learning absorption. Our research findings offer a scientific and comprehensive description of near-miss organizational learning and shed light on how to measure and improve near-miss organizational learning in the future.

**Supplementary information:**

The online version contains supplementary material available at 10.1186/s12912-022-01071-1.

## Introduction

Patient safety is an urgent and serious global public health concern. An estimated 134 million adverse events occur annually and cause 2.6 million deaths in hospitals worldwide [1]. Although learning from error is an important way to improve patient safety, only a few health organizations have achieved ideal performance. Recently, an increasing number of healthcare managers have recommended applying organizational learning for error learning to further improve patient safety [2].

A near-miss is an incident that does not reach the patient. near-misses occur more frequently than adverse events and can provide early warnings of the system’s vulnerabilities at no cost to the patient [3, 4], making them a more valuable learning source for healthcare organizations than adverse events. In addition, learning from near-misses is critical for healthcare organizations to change from traditional retrospective, passive risk management to prospective, proactive risk management[5, 6]. However, due to their salient nature, near-misses are more difficult to grasp and learn from than adverse events [7].

Despite the importance of near-miss organizational learning, most studies have been concerned with their prevalence, contributing factors, underreporting issues and quality improvement projects in healthcare organizations[8–10]. Only a few studies have attempted to explore near-misses from the organizational learning perspective, and most of them lacked the guidance of organizational learning theory[10, 11]. Other high-risk industries have developed far more advanced near-miss organizational learning than the healthcare industry, including sophisticated near-miss management systems, applied remote sensing techniques, wearable devices and data mining to increase efficiency in knowledge production, knowledge dissemination and knowledge exploitation[12]. To close this gap, it is vital to obtain a comprehensive and in-depth understanding of near-miss organizational learning in the healthcare industry and clarify future research directions.

The concept of organizational learning was first proposed in the 1960s, and the classic definition regards it as a process of detecting and correcting errors through which organizations can update their cognition and behaviour, thus improving their organizational performance [13, 14]. Organizational learning theory has flourished during the past 60 years. In particular, the 4I Framework of Organizational Learning is widely acknowledged [15]. It has been accepted in many countries across various industries, and it has been recommended for application in error learning among Chinese organizations [16, 17]. In addition, theory developers have established a corresponding measuring instrument to facilitate its implementation [18]. Thus, considering its accuracy and user friendliness, the 4I Framework of Organizational Learning was selected to guide this research [17].

In the 4I Framework of Organizational Learning, organizational learning occurs through the interaction between intuiting, interpreting, integrating and institutionalizing across the individual, group and organizational levels. Intuiting is the process of preconscious pattern recognition and pattern comprehension that influences subsequent coping behaviour. Interpretating at the individual level is the explanation through words and/or actions of an insight or idea to one’s self and others. Interpreting at the group level is the development of a shared mental model. Integrating at the group level is the process of mutual adjustment among group members and the formation of collective actions. Integrating at the organizational level is the process of synthesizing and integrating group-level learning stocks. Institutionalizing is the process of establishing well-tested knowledge into organizational memory and forming new rules and procedures. In addition, the theory developer stressed that organizational learning and its absorption have a positive relationship with organizational performance [15].

Considering the importance of near-miss organizational learning, the significant role of nursing organization in patient safety and the scarce of research in this area [19], we investigated near-miss organizational learning in a Chinese nursing organization of a tertiary hospital.

The research questions were developed under the guidance of the 4I Framework of Organizational Learning and explored in the surveyed nursing organization:

RQ1. What are the characteristics of organizational learning?

RQ2. How is the intuiting and interpreting of near-miss organizational learning at the individual level?

RQ3. How is the interpreting and integrating of near-miss organizational learning at the group level ?

RQ4: How is the integrating and institutionalizing of near-miss organizational learning at the organizational level?

## Methods

### Study design

Our research adopted a mixed methods research design for two reasons. First, according to the hypotheses of the 4I Framework of Organizational Learning [19], the survey results of the Strategic Learning Assessment Map (SLAM) can help us select representative units and explore near-miss organizational learning more efficiently. Second, through a comprehensive literature review, the researchers noted the need to apply both instruments and interviews to measure near-miss organizational learning variables. Thus, we applied an explanatory sequenced mixed methods design and followed the Good Reporting of a Mixed Methods Study (GRAMMS) to report this research (Appendix 1) [20, 21].

### Setting and participants

The study was conducted in a Chinese nursing organization of a tertiary hospital from October 2020 to January 2021. This nursing organization contains 61 nursing units and 1132 nurses. The registered nurses who worked in the front-line clinic were eligible participants. Those who worked in positions other than nursing or were on sick leave were excluded. In quantitative study, the minimum sample size was recommended to range from 285 to 291 based on the size of the organization’s population (1100–1200). With the estimation of a valid questionnaire rate of approximately 50%, the sample size was determined to be 600 [22]. The participants were identified by the staff list and recruited through simple random sampling. In the qualitative study, we enrolled participants from high-, middle- and low-scored nursing units by purposive sampling. Data collection and content analysis were conducted at the same time, and the interviews were stopped when data saturation was achieved.

### Data collection

The researchers distributed the survey to the head nurses, and they delivered the survey to selected respondents. After the completion of the survey, we applied purposive sampling to recruit participants for semi-structured interviews. We divided the surveyed nursing units into high-, middle- and low-scored groups based on the mean score of the SLAM and selected one unit as a representative in each group. Then, we interviewed nurses from various managerial levels from the selected units in the nurse station or lounge of the department. The interviewers provided informed consent, and permission was received before the interviews were audiotaped. The audiotapes were transcribed verbatim, checked for accuracy and imported into NVivo 11.0 after the completion of the interviews.

## Measures

According to the research questions, the research variables and their measurements can be seen in Table 1, and the details of the interview questions can be seen in Table 2.

**Table 1:** Research variables and their measurement

Variables	Measurement
Organizational learning	Chinese Version of Strategic Learning Assessment Map
Near-miss organizational learning performance	Near-miss learning performance at the individual level: 1.Quiz about the definition of near-misses and interview question 1 2.The Scale for Second-Order Problem-Solving Behavioural Intention following Near-misses and interview question 2 Near-miss learning performance at the group level: Quiz about near-miss reporting behaviour and interview questions 3, 4 and 5 Near-miss learning performance at the organizational level: interview questions 6 and 7

The following are the details of these measurements

### The Chinese version of the SLAM

The SLAM is an instrument built on the 4I Framework of Organizational Learning. It can offer an overall description of organizational learning and diagnose existing problems [19]. It contains six domains: individual-level learning stocks (II), group-level learning stocks (GG), organizational-level learning stocks (OO), feed-forward learning flows (FF), feed-back learning flows (FB) and organizational performance (PERF). Each domain has 10 items. All items are rated on a 7-point Likert scale (1 = strongly disagree, 7 = strongly agree); the higher the score is, the better the performance. In addition, we can use an equation to calculate misalignment, which can reflect learning absorption. The equation is Misalignment= (‾*x*_II_+‾*x*_GG_+‾*x*_OO_)/3-(‾*x*_FF_+‾*x*_FB_)/2. This instrument has been used in North America and European countries [16, 19]. Our research team obtained permission for its cross-cultural adaptation and adapted it to Chinese followed the Recommendations for the Cross-Cultural Adaptation of the DASH & Quick DASH [23]. Besides, the theoretical hypotheses of the 4I Framework of Organizational Learning have been verified in nursing organizations among Chinese tertiary hospitals. The Cronbach’s α for the overall scale and the sub-scales of the Chinese version ranged from 0.97 to 0.99, the I-CVI was 0.87, and the S-CVI/Ave was 0.98 [24].

### Quiz about near-miss definition

To measure nurses’ recognition of near-misses, the researchers developed an item that asked them to select the correct definition among four choices, including definitions of near-misses, adverse events, hazards and sentinel events. The higher the accuracy, the better the intuition [19] .

### The Scale for Second-Order Problem-Solving Behavioural Intention following Near-misses

Intention for behaviours following near-misses can be grouped into two categories: the intention for first- and second-order problem-solving behaviour following near-misses, among which only second-order problem-solving behaviour can promote near-miss organizational learning and reduce future risk. The researchers applied the Scale for Second-Order Problem-Solving Behavioural Intention following Near-miss to test nurses’ intention of near-miss coping behaviours. Its S-CVI/Ave is 1.0, the overall Cronbach’s α is 0.909, and the Cronbach’s α for its sub-scales ranges from 0.799 to 0.875. It contains five dimensions: reporting intention, sharing intention among colleagues, intention for cause exploration, practice changing intention and continuous improvement intention. It has 23 items, and all items are rated on a 5-point Likert scale, from 1 = strongly disagree to 5 = strongly agree. The higher the score, the more likely nurses are to engage in second-order problem-solving behaviour [25].

### Problem-solving behaviours following near-miss

It is widely acknowledged that problem-solving behaviours generally include first- and second-order problem-solving behaviours. First-order problem-solving behaviour occurs when nurses perform a quick fix and continue to finish a task that is blocked or interrupted, whereas second-order problem-solving behaviour occurs when nurses perform a quick fix, take action to address underlying causes and bring the problem to managers’ attention. Generally, error reporting can be regarded as a sign of second-order problem-solving behaviour [26]. The researchers evaluated reporting behaviour following near-misses by a self-reported item borrowed from the Hospital Survey on Patient Safety Culture [27]: ‘When a mistake is made in your hospital work area/unit but is caught and corrected before affecting the patient, how often is this reported?’ The participants could choose never, rarely, sometimes, most of the time, or always to describe their reporting behaviours. Their choice reflected their preference for different types of problem-solving behaviour.

### Semi-structured interview guideline

Qualitative interviews were jointly performed through individualized interviews and focus group interviews to avoid power differentials and their negative influence. The interview guideline (Table 2) was developed based on the 4I Framework of Organizational Learning. The researchers conducted individualized interviews with nurse leaders (*n* = 4) and three focus group interviews with 12 nurses at the non-managerial level.

**Table 2:** Semi-structured interview guideline

Domain	Semi-structured questions
Individual-level learning	Q1: Are you familiar with near-misses, and what is your comprehension? Q2: What would you do if you encountered a near-miss in your work? How about your colleagues? Q3: Have you ever reported a near-miss?
Group-level learning	Q4: How does your nursing unit deal with near-misses? Q5: Are there any regulations?
Organizational-level learning	Q6: How does your nursing organization deal with near-misses? Q7: Are there any regulations?

## Data analysis

### Statistical analysis

#### Quantitative data analysis

Quantitative data were imported and analysed by SPSS 24.0. Means, standard deviations and percentiles were used for statistical description. The hypothesis testing of the SLAM was analysed by Structural Equation Modelling (SEM) using AMOS 25.0. In addition, the alpha level was set at 0.05.

**Qualitative analysis**: Qualitative data were analysed using content analysis (deductive and inductive) by two independent researchers with the following steps: [1] an analysis matrix was built under the 4I Framework of Organizational Learning (see Appendix 2); [2] the researchers reviewed the transcribed text thoroughly and repeatedly to become familiar with the text content; [3] the researchers analysed and compared their coding with the initial three interviews and then discussed the discrepancies and developed the initial version of the codebook; [4] the researchers analysed the following interviews and categorized all the codes into the analysis matrix; [5] the researchers went through the text again and conducted inductive content analysis to determine whether there were new codes or categories that should be added and then determined the final version of the codebook [28].

### Integration of data and emergent themes

To integrate the qualitative and quantitative data, the findings are displayed together and merged under the analysis matrix.

### Validity and reliability/rigor

We applied various methods to guarantee the credibility, dependability, confirmability and transferability of our research [29].

#### Credibility

① All of the researchers had experienced with quantitative studies and qualitative studies, thus ensuring the credibility of our research. ② In this research, we applied both quantitative and qualitative research to investigate near-miss organizational learning (methodological triangulation); in addition, our data were collected from various sources, including surveys, interviews and records in the Adverse Event Reporting System (data triangulation). Furthermore, two researchers participated and discussed the coding until a consensus was reached during content analysis (investigator triangulation).

#### Dependability & confirmability

The researchers recorded every research procedure and all the details, and the research members checked these audits regularly.

#### Transferability

The researchers provided detailed information about the nursing organization as well as the theory and instruments applied in this survey. Therefore, risk managers in similar healthcare organizations can easily apply our research results.

### Ethical considerations

The study was reviewed and approved by the Ethics Committee of the School of Nursing, Chinese Academy of Medical Sciences & Peking Union Medical College (IRB approval number: 201,902). All participants voluntarily participated in this research, and we obtained their informed consent before the survey and interview.

## Results

### Characteristics of nursing organizations and participants

A total of 560 nurses from 46 nursing units completed the SLAM, and 376 valid questionnaires were analysed. There were 341, 33 and 2 nurses from the non-, middle- and high-managerial levels, and their length of employment was 8 [4, 11] years. A total of 598 nurses completed the second survey, including the Scale for Intention of Second-Order Problem-Solving Behaviour following near-misses, the definition and reporting behaviours for near-misses. There were 349 valid questionnaires in the second round. The participants’ length of employment in this round was 10 (5, 12.75) years, and their length of employment in their nursing unit was 7 [3, 10] years.

## Organizational learning

### Score of organizational learning

As shown in Table 3, the mean score of every dimension in the SLAM was less than 6 (6 = agree), higher than that of previous studies but still showing room for improvement [16, 19]. Among all the dimensions, the individual-level learning stocks scored the highest, and the feed-forward learning flows scored the lowest.

**Table 3:** Survey results of organizational learning (*N* = 376)

Dimension	Mean (SD)
Individual-level learning stocks	5.85(0.93)
Group-level learning stocks	5.83(0.91)
Organizational-level learning stocks	5.76(0.91)
Feed-forward learning flows	5.74(0.92)
Feed-back learning flows	5.77(0.95)
Organizational performance	5.59(0.94)
Misalignment	0.06(0.41)

### Characteristics of organizational learning

**Fig. 1:**
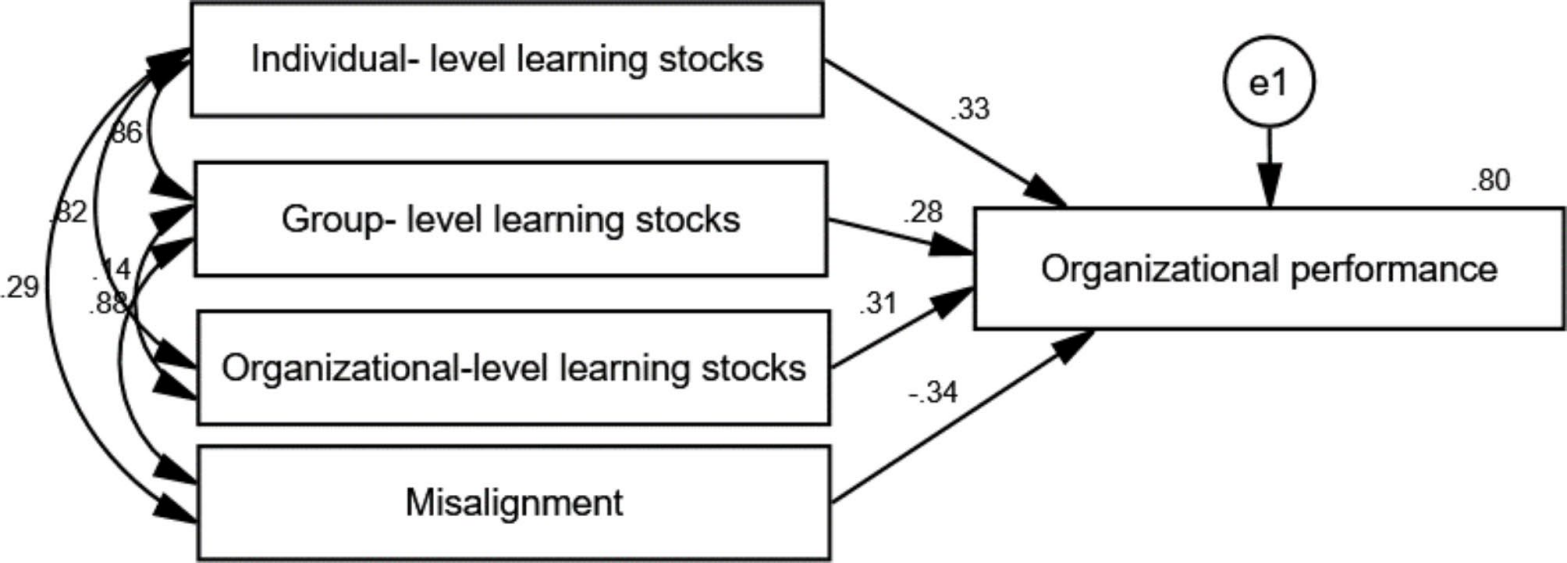
Characteristics of organizational learning in surveyed nursing organizations

The SEM results verified the 4I Framework of Organizational Learning, indicating that individual-, group- and organizational-level learning stocks have a positive relationship with organizational performance and that misalignment has a negative relationship with organizational performance (Fig. 1). The model-fit indices were χ^2^ = 0.775, *p* = 0.379, RMSEA < 0.01.

The regression equation is organizational performance = −0.772 misalignment + 0.331 individual-level learning stocks + 0.290 group-level learning stocks + 0.322 organizational-level learning stocks+ 0.15, and the standardized regression coefficients are as follows: *β*_II_ = 0.334, *β*_GG_ = 0.284, *β*_OO_ = 0.313, and *β*_Misalignment_=-0.339.

### Performance of near-miss organizational learning

#### Near-miss recognition

Only 33% of the surveyed nurses correctly recognized near-miss, and most of them regarded them as sentinel event (8%), adverse event (17%) or hazard (42%).

#### Second-order problem-solving behaviour intention following near-misses

Among all the dimensions, reporting intention scored lowest, indicating that the respondents had a stronger intention towards first-order problem-solving behaviour in their work (Table 4).

**Table 4:** Second-Order Problem-Solving Behaviour Intention following Near-misses (*n* = 349)

Dimension	Mean (SD)
Practice changing intention	4.20(0.59)
Sharing intention among colleagues	4.17(0.53)
Intention for cause exploration	4.22(0.53)
Reporting intention	4.13(0.65)
Continuous improvement intention	4.22(0.56)

#### Reporting behaviours for near-misses

**Fig. 2:**
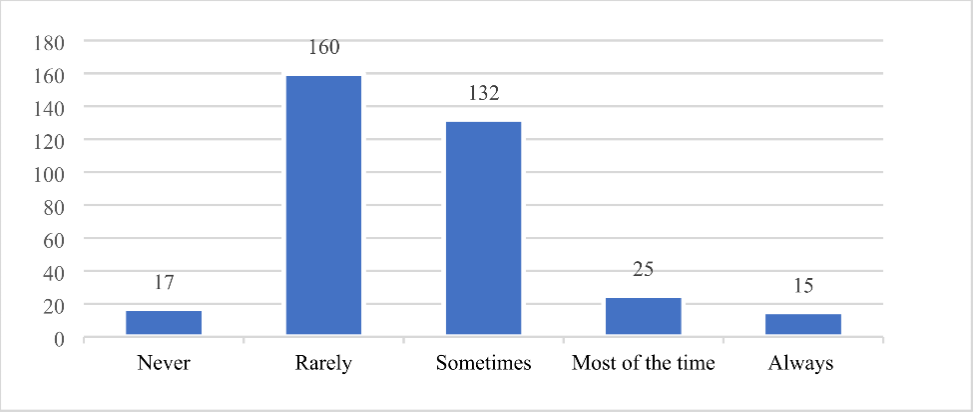
Reporting behaviours towards near-miss

A total of 50.7% of the surveyed nurses indicated that they rarely or never reported near-misses to the Adverse Event Reporting System (Fig. 2). This result is consistent with their stronger intention of first-order problem-solving behaviour.

### Coding of near-miss organizational learning

In accordance with the mean score of SLAM, we divided the 46 surveyed nursing units into a high-scored group (*n* = 12), a middle-scored group (*n* = 21) and a low-scored group (*n* = 13). Then, we selected one nursing unit in each group for the interview. These nursing units were department of gynaecology, emergency department and department of urology. We recruited 16 nurses across different managerial levels within the nursing organization, among which there were 1, 5, 4 and 6 nurses from the nursing department, department of gynaecology, emergency department and department of urology, respectively. We conducted interviews until data saturation, and the length of the interviews ranged from 20 to 45 min. After content analysis, we developed 5 themes, 9 categories and 13 codes to describe near-miss organizational learning (Appendix 2).

### Integration of data and emergent themes

The researchers merged the quantitative and qualitative research results under the analysis matrix (Table 5), through which we can see that the quantitative and qualitative research results describe near-miss organizational learning in a similar way but enrich each other.

**Table 5:** The integration of data and emergent themes

Theme	Category	Quantitative Data	Code
Individual-level learning	Intuiting	33% of respondents recognized near-misses correctly	Unfamiliarity with near-misses
		The intention of reporting scored the lowest among the dimensions (4.13 ± 0.65)	Stronger intention of first-order problem-solving behaviour
	Interpreting	50.7% of respondents indicated that they never or rarely reported near-misses to the Adverse Event Reporting System There were only 22 records of near-misses in the Adverse Event Reporting System in 2020	Dominance of first-order problem-solving behaviour
Group-level learning	Interpreting	Group-level learning contributes the least to organizational performance (*β*_GG_ = 0.284)	Unsystematic near-miss learning in the nursing unit
	Integrating		Lack of evaluation and recording of near-miss learning in the nursing unit
Organizational-level learning	Integrating	The mean score of FB10 (When making decisions for the future, we do not seem to have any memory of the past) was lowest among all items of the Strategic Learning Assessment Map (‾*x* = 4.90)	Lack of integration of learning stocks among different nursing units
	Institutionalizing		Lack of standardized near-miss management documentation
			Nonexistence of the institutionalizing work of near-miss organizational learning
Feed-forward learning	Rare feed-forward learning	Large misalignment (*β*_misalignment_=−0.339)	Suspension of near-miss organizational learning from the group level
Feed-back learning	Inconsistent comprehension of near-miss management		No need to report at the individual level
			No need to report at the group level
			Required to report at the organizational level
	Poor utilization of near-misses in improving patient safety		No feed back towards near-miss learning

## Discussion

It is widely acknowledged that near-misses play an important role in the transformation of risk management in healthcare organizations [30, 31], and organizational learning has been recommended as a promising strategy for patient safety [2]. However, only a few researchers have explored near-miss organizational learning, among which most have explored certain aspects or lacked a theoretical foundation [32–34]. Our research investigated near-miss organizational learning with a sound theoretical foundation and valid instruments, provided comprehensive and deep insight into near-miss organizational learning and offers suggestions for future improvement.

### Characteristics of organizational learning

First, our research results supported the theoretical hypotheses of the 4I Framework of Organizational Learning in the surveyed nursing organizations, indicating that individual-, group- and organizational-level learning have a positive relationship with organizational performance and that misalignment has a negative relationship with organizational performance. Based on this assumption, we divided the 46 surveyed nursing units into low-, middle- and high-scoring groups and continued to investigate near-miss organizational learning performance, thus ensuring the accuracy, comprehensiveness and efficiency of our research [35]. Second, the group-level learning stocks contributed the least to organizational performance, and the misalignment was much larger than that of previous studies and played a negative role in organizational performance (*β*_Misalignment_= -0.339) [16, 19]. These two main features indicate that near-miss organizational learning may be hampered at the group level and that the weakness in group-level learning may be an important reason for the unsatisfactory performance of patient safety management, which coincides with another study on error learning among healthcare organizations [36]. Therefore, organizational learning behaviour may be universally problematic and needs to be addressed to better improve the quality and safety of patient care.

### Near-miss organizational learning performance

#### Individual-level learning of near-misses

The quantitative and qualitative results indicate that nurses have difficulty recognizing near-misses. Although they consider near-misses important for patient safety, most of them cannot tell the difference between near-misses, adverse events and hazards, and this confusion negatively influences nurses’ intentions and coping strategies. Our research finding is consistent with that of other studies [9, 37, 38]. The main reasons for unfamiliarity with near-misses include the inadequacy of emphasis on near-miss management and inconsistency in near-miss definitions in Chinese healthcare organizations [39, 40]. The research results also exhibited an obvious preference for first-order problem-solving behaviour among nurses over second-order problem-solving behaviour. This finding is similar to that of other studies both in China and abroad [9, 37, 38]. Thus, the preference for first-order problem-solving behaviour prevails in most nursing organizations and poses a threat to near-miss organizational learning [41]. In addition, the nurses stated that they did not always report near-misses to the Adverse Event Reporting System; thus, first-order problem-solving behaviour dominates. Finally, unlike organizations in other high-risk industries, most healthcare organizations in China do not have a separate near-miss reporting system; thus, nurses may believe that reporting near-misses may damage their reputation and bring them shame and criticism [9, 12, 42]. Fortunately, with the increasing awareness of the importance of near-misses, an increasing number of healthcare managers and researchers have advocated a separate near-miss reporting system [37, 43].

### Group-level learning of near-misses

The suspension of learning from the group level is eye-catching and can be supported by the unsatisfactory performance at the group level in the organizational learning model (*β* = 0.284) and nurses’ descriptions of their regulation and management of near-misses in their units. Most interviewees said they never reported near-misses at the group level, and they had no systematic plan for analysing, ameliorating and evaluating near-misses in their unit. In addition, there was not always feedback to group members. In addition to the above-mentioned research findings, we found that although nurses sometimes performed actions and accumulated valuable experiences, these near-miss learning stocks were not well documented. For example, they were inclined to share near-misses in WeChat to keep their colleagues alert, but this method cannot facilitate the formation of organizational memory and is difficult to consult when needed. This research result not only indicates the detailed problems in group-level learning for near-misses but also identifies the reasons, such as a lack of near-miss management regulations, which lead to inconsistency in near-miss comprehension, stronger intention and inclination towards first-order problem-solving behaviour, and a lack of clarity when handling near-misses, making subsequent integration and institutionalization difficult to achieve.

### Organizational-level learning of near-misses

Due to the suspension of near-miss learning at the group level, it is very difficult to continue near-miss organizational learning. The surveyed nursing organizations have few group learning stocks to integrate; in fact, there were only 22 near-miss records in the Adverse Event Reporting System in 2020 [44], similar to another study (25 near-misses per year) [7]. Thus, this system is incapable of reflecting the real situation and facilitating prospective, proactive risk management. Even worse, the institutionalization of near- miss organizational learning is missing.

### Problems in feed-forward and feed-back learning

Although it is required to report near-misses to the Adverse Event Reporting System based on regulations, most nurses did not comply with this regulation. The researchers noted contradictory perspectives on near-miss reporting protocols at the individual, group and organizational levels; this inconsistency in near-miss cognition also exists in other industries [12]. These two features indicate poor feedback learning for near-misses in this nursing organization, which damages organizational learning [19]. Nurses’ stronger intention and more frequent performance of first-order problem-solving behaviour hampers feed-forward learning; thus, current near-miss knowledge cannot be refined and stored as organizational memory.

## Implication

Our research paves the way for measuring near-miss organizational learning following the 4I Framework of Organizational Learning by identifying variables across all the levels in the organization and providing valid instruments and interview guidelines, thus facilitating the diagnosis and improvement of near-miss organizational learning in both nursing organizations and other healthcare organizations. Considering the similarities in near-miss organizational learning behaviour [24], the problems in near-miss organizational learning identified in this study may also exist in other nursing organizations in Chinese tertiary hospitals. The proposed suggestions for future improvement are also meaningful for these hospitals.

## Limitations

There are some limitations in our research. First, we only conducted the survey and interview in one nursing organization in a Chinese tertiary hospital; thus, we should interpret the results with caution. In addition, most of the outcome indicators were assessed subjectively through retrospection; thus, our results may be influenced by recall bias and social approval tendency [45–47).

## Conclusion

Although near-miss organizational learning is important for prospective, proactive risk management, our research determined that near-miss organizational learning is much more difficult than expected. The current organizational learning behaviour is not conducive to near-miss organizational learning, and we identified the multilevel learning behaviours and characteristics of near-miss organizational learning. In addition to traditional individual-level or organizational-level learning for near-misses, group-level learning is a key factor as well as a weakness that should be emphasized in the future.

## Electronic supplementary material

Below is the link to the electronic supplementary material.

Supplementary Material 1

Supplementary Material 2

## Data Availability

All data generated or analyzed during this study are included in this published article [and its supplementary information files].
