# Supplementary material for: Near-miss organizational learning in nursing within a tertiary hospital: a mixed methods study
Source: BMC Nurs. 2022 Nov 16;21:315. doi: 10.1186/s12912-022-01071-1 (PMC9667619; doi:10.1186/s12912-022-01071-1)
Supplement: Supplementary file 1 — Supplementary Material 1 [file 12912_2022_1071_MOESM1_ESM.docx]

**Appendix 2:**

**TABLE 1 The analysis matrix**

| **Theme** | **Category** | **Code** | **Illustrative quotation** |
| --- | --- | --- | --- |
| Individual-level learning | Intuiting | Unfamiliarity with near miss | I think near miss is hazard actually(N1) |
|  |  | Stronger intention of the first-order problem solving behaviour | Generally, if we made mistake but it did not reach the patient, we will fix it immediately, we only reported it unless we cause some harms to the patient. (N3) |
|  | Interpreting | Dominance of the first-order problem solving behaviour | In most cases, we did not report it to the Adverse Event Reporting System, not to mention the following analysis, because it did not cause any harm to the patient and we are too busy in our work. (N10) |
| Group-level learning | Interpreting | Unsystematic near miss learning in the nursing unit | We have heard about it sometimes, but it seemed that we didn’t have standard regulation to guide our near miss management in our nursing unit and prevent the future occurrence of similar near miss. (N15) |
|  | Integrating | Lack of evaluation and recording of near miss learning in the nursing unit | According with my experience, we only conduct systematic work of evaluation and recording for adverse event in our unit. (N8) |
| **TABLE 1 The analysis matrix (Continue)** | | | |
| **Theme** | **Category** | **Code** | **Illustrative quotation** |
| Organizational-level learning | Integrating | Lack of integration of learning stocks among different nursing units | In our patient safety meeting, we seldom share near miss and its lessons among different units in our hospital. (N8) |
|  | Institutionalizing | Lack of standardized near miss management document | Mostly, we delt with near miss based on our working experience, when you reported near miss to head nurse, she will ask you to solve it by yourself. (N11) |
|  |  | Nonexistence of the institutionalizing work of near miss organizational learning | I have no impression of regulations for near miss in our nursing organization. (N16) |
| Feed-forward learning | Rare feed-forward learning | Suspension of organizational learning about near miss since the group level | The management of near miss is always stopped at the unit level, since it did not cause any harm to the patient. (N3) |
| Feed-back learning | Inconsistent comprehension towards near miss management | No need to report at the individual level | At present, it is not required to report near miss to the Adverse Event Reporting System. (N5) |
|  |  | No need to report at the group level | Since we have prevented it from reaching the patient, thus our unit did not report it to the Adverse Event Reporting System. (N10) |
| **TABLE 1 The analysis matrix (Continue)** | | | |
| **Theme** | **Category** | **Code** | **Illustrative quotation** |
| Feed-back learning |  | Required to report to the organizational level | For these near misses, we also required to report, you have made a mistake, and it can offer early warning to the system and experiences for other units. (N1) |
|  | Poor utilization of near miss in patient safety improvement | No feed back towards near miss learning | There will be two sessions of patient safety meeting in our hospital a year, but they only share typical cases of adverse event and their lessons. (N9) |
