# Supplementary material for: Near-miss organizational learning in nursing within a tertiary hospital: a mixed methods study
Source: BMC Nurs. 2022 Nov 16;21:315. doi: 10.1186/s12912-022-01071-1 (PMC9667619; doi:10.1186/s12912-022-01071-1)
Supplement: Supplementary file 2 — Supplementary Material 2 [file 12912_2022_1071_MOESM2_ESM.docx]

**The Good Reporting of a Mixed Method Study(1)**

| **Item No** | | **Item description** | **Page No.** |
| --- | --- | --- | --- |
| **Domain 1: Describe the justification for using a mixed methods approach to the research question** | | | |
| 1a | | Justification for including quantitative design | 4 |
| 1b | | Justification for including qualitative design | 4 |
| 1c | | Justification for using a mixed methods approach | 4 |
| **Domain 2: Describe the design in terms of the purpose, priority and sequence of methods** | | |  |
| 2a | | Purpose | 4 |
| 2b | | Priority | 4 |
| 2c | | Sequence | 4 |
| **Domain 3: Describe each method in terms of sampling, data collection and analysis** | | |  |
| Quantitative design | 3a | Sampling | 4 |
|  | 3b | Data collection | 4 |
| **Item No**  **The Good Reporting of a Mixed Method Study(1) （Continued）** | | **Item description** | **Page No.** |
| Quantitative design | 3c | Analysis | 7 |
| Qualitative design | 3d | Sampling | 4 |
|  | 3e | Data collection | 4 |
|  | 3f | Analysis | 7 |
| **Domain 4: Describe where integration has occurred, how it has occurred and who has participated in it** | | | |
| 4a | | Where integration has occurred | 7-8 |
| 4b | | How integration has occurred | 7-8 |
| 4c | | Who has participated in integration | 7-8 |
| **Domain 5: Describe any limitation of one method associated with the present of the other method** | | | |
| 5 | | Limitation of one method associated with the present of the other method | 4 |
| **Domain 6: Describe any insights gained from mixing or integrating methods** | | | |
|  | | Insights gained from mixing or integrating methods | 14-17 |

1.Cameron R, Dewyer T, Richardson S, Ahmed E, Sukumaran A. Lessons from the field: applying the good reporting of a mixed methods study (GRAMMS) framework. Electronic Journal of Business Research Article. 2013; 11:55-66.
